# Conservative Media Use and COVID-19 Related Behavior: The Moderating Role of Media Literacy Variables

**DOI:** 10.3390/ijerph19137572

**Published:** 2022-06-21

**Authors:** Porismita Borah, Kyle Lorenzano, Anastasia Vishnevskaya, Erica Austin

**Affiliations:** 1 Edward R. Murrow College of Communication, Washington State University, Pullman, WA 99163, USA; a.vishnevskaya@wsu.edu (A.V.); eaustin@wsu.edu (E.A.); 2 School of Communication, Film and Media, University of West Georgia, Carrollton, GA 30118, USA; kyle.lorenzano@gmail.com

**Keywords:** COVID-19 protective behavior, media literacy for content, media literacy for source, conservative media use

## Abstract

At the start of the COVID-19 pandemic, there was no vaccine to cure or slow its impact due to the novelty of the virus, nor were there were any other standardized measures to handle its spread. Yet, despite the detrimental consequences of the pandemic and its impact on people’s lives, the behavior of individuals to combat the pandemic was not necessarily consistent with official guidelines. To make things worse, the pandemic was highly politicized in countries such as the U.S. With a help of a national survey from the U.S., we examine the associations between media literacy variables and willingness to perform recommended COVID-19 related health behavior. Moreover, we also examine the moderating role of conservative media use in this relationship. Our findings show that conservative media use was negatively associated with these protective behaviors, and that both media literacy variables were positively related with willingness to perform recommended COVID-19 related health behavior. Our results show that media literacy can mitigate some of the impact of conservative media use on individuals. Our findings help understand the complexity of protective behavior against the virus during a highly politicized pandemic.

## 1. Introduction

The first case of COVID-19 was registered in Wuhan, China, at the end of November 2019 [1]. The virus eventually spread out globally by the mid-spring 2020 [2], and the United States had the first COVID-19 case confirmed earlier in January of 2020 (e.g., [1]), around the same time as the human-to-human transmission of the virus was officially confirmed [1,3]. By the summer of 2020, as reported globally, the virus resulted in more than 400,000 deaths world-wide [4] and more than 100,000 deaths in the United States [5]. On top of the impact that the virus had on the death toll world-wide, COVID-19 affected social [6], ecological [7], political [8] and economic [9] spheres of activities across the globe [10].

At the start of the pandemic, there was no vaccine to cure or slow its impact due to the novelty of the virus, nor were there any other standardized measures to handle its spread [11]. Alongside the World Health Organization officially declaring the spread of the virus as a global pandemic in March 2020 [12], the CDC recommended multiple measures to combat or restrict the virus while the vaccine was still being developed [1]. These measures included maintaining a minimum distance of six feet during social interactions, refraining from group gatherings, and avoiding large social gatherings and/or meetings [12]. The United States also declared a nation-wide state of emergency that included “stay-at-home” orders [13]. Various public organizations and institutions implemented additional preventive measures such as mask mandates (e.g., [14]), more frequent hand washing with soap and water (e.g., [15], travel restrictions (e.g., [16]), and physical distancing (e.g., [6]). Yet, despite the detrimental consequences of the pandemic and its impact on people’s lives, the behavior of individuals to combat the pandemic was not necessarily consistent with these guidelines.

To make matters worse, the pandemic was highly politicized in countries such as the U.S. [17]. Moreover, conservative media have amplified these political differences. Throughout the pandemic, conservative media sources such as Fox News often understated the impact of the virus [18]. These sources often called the pandemic a “hoax” or “fraud” (e.g., [19]). These media sources also discussed how the COVID-19 pandemic was an excuse to impeach Trump [20]. In this situation, media literacy may play a protective role and help in decision-making [21,22]. In the current study, we use a national survey from the U.S. to examine the associations between media literacy variables and willingness to perform recommended COVID-19 related health behavior. Moreover, considering the politicization of the pandemic, we also examine the moderating role of conservative media use in the relationship between media literacy and willingness to perform recommended COVID-19 related health behavior. Our findings speak to the complexity involved in deciding to adopt protective behaviors against the virus during a highly politicized pandemic.

### 1.1. COVID-19 Pandemic and Behavior

As existing studies show, there are numerous factors that can explain peoples’ behavior related to the pandemic. For instance, recent research [23] discuss the psychological factors that determine preventive COVID-19 behavior patterns among the population. These preventive COVID-19 behaviors included social distancing, mask wearing, and respiratory hygiene practices, such as washing hands or coughing into a tissue. They found that the perceived effectiveness of such measures is a strong predictor of compliance with those behaviors. With respect to psychological factors, perceived loss of freedom in relation to preventative behaviors has been found to be negatively associated with mask-wearing, while conflict avoidance has shown a positive association with willingness to wear a mask [24]. Demographic factors also had an effect on safety behavior with regard to COVID-19 [25]. Other studies have found that female participants were more likely to wear masks, while Caucasian respondents were associated with less mask-wearing and less adoption of social distancing practices [25].

A recent study [26] analyzed the role of COVID-19-related news watching on behavior compliance and found that exposure to COVID-19-related news is important for safety behavior compliance. While research shows [26] that watching and seeking COVID-19-related news affects safety behavior compliance, it should be noted that the news coverage on COVID-19 has been politicized and also contained misinformation [27]. Moreover, messages from the former President Donald Trump undermined the authority of the health experts and contributed to undermining the national unity around combating the pandemic [17]. However, COVID-19 misinformation is one among several other factors that contributed to the public’s mental health morbidity [28]. With this in mind, the importance of people’s cognitive characteristics, and their ability to identify, access, and process reliable information becomes even more significant [10]. Existing scholarship suggests that media literacy is one of the tools that can be used to combat misinformation in the media and its effects [29]. While epidemiological information did not effect safety behavior compliance, the political ideology of the participants did. They found that liberal participants tended to comply with safety policies and rules more than conservative individuals [30]. 

### 1.2. Role of Media Literacy

Past research seems to suggest that media literacy may be positively associated with behaviors such as COVID-19 health behavior (e.g., [31,32]). When referring to media literacy, scholars usually perceive the concept as the spectrum of knowledge that allows individuals to “navigate the complex news and information environment” [33], p. 1. Media literacy also involves the ability of the message recipients to properly interpret media messages (e.g., [34]). Research on media literacy suggests that the ability to acquire and critically analyze information affects people’s beliefs (e.g., [35]), attitudes and behavioral intentions (e.g., [36]), political efficacy (e.g., [37]), and perceptions (e.g., [32]). Studies also show that higher levels of literacy are associated with lower levels of fear [22] and showed protective effects against COVID-19-related depression [38]. As media literacy involves the ability to think critically while processing information, it also helps to facilitate decision making [39]. Increased literacy not only facilitates the differentiation of factual information from misinformation, but also allows people to make better, more informed health decisions as well as practice safer and healthier behaviors (e.g., [31]).

Previous studies have also demonstrated media literacy’s association with shaping individuals’ beliefs surrounding COVID-19. Recent evidence [40] suggests that, while incidental news exposure is associated with COVID-19-related misperceptions, an individual’s own confidence in their media literacy skills (i.e., self-perceived media literacy) can mitigate incidental exposure’s association with these same misperceptions. Other constructs associated with media literacy, such as need for cognition and one’s own perception of control of the information consumed (i.e., media locus of control), have been found to have both direct and indirect associations with decreased misperceptions of COVID-19 [41]. Outside of prior research on media literacy generally, further research on health-specific media literacy skills have revealed that those with higher digital health literacy are more likely to report intention to vaccinate and take the prospect of COVID-19 infection seriously [42]. At the same time, additional research has suggested that there are limits to the effectiveness of media literacy when accounting for political ideology, such that it can serve to reduce misperceptions among self-identified liberals but not among conservatives under the same circumstances [40].

In the current study we use two literacy variables, “media literacy for source of news” and “media literacy for content of news” [43], as moderating variables. The moderator involving the ‘source of news’ involves thinking critically about the source attributed to news and information, while the ‘content of news’ moderator is centered around critically thinking about the news content itself [43]. Thus, we propose the following hypotheses:

**Hypothesis** **1 (H1).** *Media literacy of source will be positively associated with willingness to perform recommended COVID-19 health behavior*.

**Hypothesis** **2 (H2).** *Media literacy of content will be positively associated with willingness to perform recommended COVID-19 health behavior*.

### 1.3. Politicization of COVID-19

To the extent that U.S. politics have been marked by partisan and ideological disagreement during the last several decades [44], much of the existing literature on the subject suggests that increasing polarization is driven by elites [45]. However, the COVID-19 pandemic presents a real time case study of a public health crisis that, in theory, would warrant an apolitical response. Existing literature points to there being clear partisan differences in adherence to social distancing and/or shelter-in-place orders [46,47,48]. Research [49] found that states with Republican governors were slower to announce social distancing mandates. At the individual level, use of GPS data to track individual mobility and travel to essential vs. non-essential locations early in the pandemic has revealed that counties that voted disproportionately for Trump in the 2016 U.S. Presidential Election were both less likely to stay socially distanced and to reduce travel to non-essential locations [50]. Similar findings were corroborated [51] in that so-called ‘Trump counties’ were less likely to follow shelter-in-place orders and that this effect increased over time, despite the fact that aggregate travel decreased significantly when COVID-19 was first reported as a large-scale public health crisis.

Furthermore, in this same study, support for Trump in general and the trajectory of Trump’s public comments about the pandemic (e.g., varying between highlighting and downplaying the severity of COVID-19) was also found to have an impact on adherence to shelter-in-place orders—namely, Trump taking a more serious public tone about the virus was found to result in a smaller gap between ‘red’ and ‘blue’ counties’ mobility, while Trump downplaying the severity of COVID-19 resulted in the inverse effect. Research also shows [48] an indirect effect of conservatism on COVID-19-related behaviors such that it was associated with less likelihood of perceiving the virus as a serious threat, or that behaviors could do anything to mitigate the risks of infection, which in turn resulted in less adherence to shelter-in-place orders and therefore less reliance on contactless shopping services (relative to self-identified liberals). Results from a series of online experiments [52] have shown similar findings in that conservatives were predisposed to perceiving COVID-19 as less of a risk to their health and having a higher threshold for maximum acceptable levels of risk during the pandemic. Research [24] has also revealed that favorability towards Trump, as well as antipathy towards Joe Biden, has been both directly and indirectly associated with less adherence to mask-wearing.

Public statements by Trump and other high-profile conservative figures have also had a clear impact on both public behavior and public opinion throughout the course of the pandemic. As shown previously [53], in the absence of publicly partisan statements disputing the existence/severity of COVID-19 and effective responses to mitigating risks to public health, there is broad public consensus around social distancing guidelines. When it comes to beliefs about the nature of COVID-19, and specifically misinformation having to do with the pandemic, conservative users on Twitter have been found to amplify or even endorse COVID-19-related misinformation more often than their left-leaning counterparts [54]. Concerning the theory that the politicization of the pandemic is elite driven, it is also worth noting that elites in the media can likewise have a similar role to play alongside the president and other high profile elected officials.

With respect to public opinion and reaction to the pandemic, existing research indicates that the politicized nature of COVID-19 is reflected in the attitudes, beliefs, and behaviors of everyday citizens. As demonstrated [55], conservative ideology has been linked to less concern about being infected by COVID-19 and less adoption of protective health behaviors, although The authors find evidence that these outcomes are partly driven by a lack of trust in science (which conservatism was disproportionately associated with). In this same study, the authors note that these association are not unique to the U.S., yet are most pronounced in the U.S. and Canada [55].

### 1.4. Conservative Media Use and COVID-19 Related Behavior

Across a wide range of contemporary research on both political ideology and news media use as it relates to COVID-19, evidence suggests that reliance on conservative media is associated with lower adoption of behavioral measures aimed at mitigating risk of infection [56,57,58,59,60,61]. Existing studies show some inconsistency in the precise effect of conservative ideology or Republican partisan affiliation and its impact on COVID-related behavior, with some finding that conservatives/Republicans were less likely to adopt preventative behaviors [58] and others finding no significant relationship between the two [56]. Yet overall, the impact of conservative media appears relatively consistent in the literature.

Across these studies, a particular emphasis has been put on Fox News and its impact on preventative measures like social distancing and mask-wearing. Much like aforementioned studies on ‘Trump counties,’ recent work [56,58] suggest that exposure to Fox News led to a smaller decrease in travelling during the first lockdown mandates in March/April 2020. Additional research suggests that Fox viewers are also less likely to wear masks and be concerned for the safety of their family in response to the ongoing pandemic [59]. When considering Fox alongside other conservative news sources like the Wall Street Journal, Sean Hannity, and Brietbart, research [57] found that conservative media use was negatively associated with a series of measures representing perceived efficacy of behavior meant to prevent infection, as well as beliefs about perceived severity and susceptibility to the virus. With the exception of perceived susceptibility to the virus, Chung and colleagues also found a similar effect for Trump’s COVID-19 briefings. Further evidence [61] shows that conservative media use is negatively associated with mask-wearing as well as trust in the WHO and CDC, although they did not find a significant relationship between right-leaning sources and greater perceived risk of COVID-19.

Along with behaviors like social distancing, mask-wearing, and handwashing, conservative media has also been found to be associated with various beliefs about COVID-19, some of which could indirectly impact behavior such as one’s intent to be vaccinated. For example, in a two-wave study [62], first at the start of the pandemic in March 2020 and again in July 2020, researchers found that heavy use of conservative media was associated with belief in COVID-19-related conspiracies, which was associated with lower intention to get vaccinated. This effect grew stronger in July 2020 as progress towards developing the vaccine grew considerably from the start of the pandemic. These findings have also been corroborated [63] via content analysis that conservative media disproportionately adopted message frames associated with controversy and conspiracy theories such as the ‘Wuhan lab’ theory or the Chinese Communist Party’s culpability in the pandemic. Recent work [64] has also found that avoidance of conservative media and positive attitudes towards vaccines generally is associated with greater intent to receive the COVID-19 vaccine. However, conditional effects of conservative media use in relation to message framing were found in this same study, such that individuals who consumed high amounts of conservative media were relatively more likely to indicate vaccine intention when messaging highlighted one’s own benefit of receiving the COVID-19 vaccine rather than its benefits for others in their surrounding community. Outside of the U.S. media and political context, research on COVID-related Facebook pages in Brazil indicates that links to information and particularly user posts on right-wing pages were a greater source of disinformation, whereas left-wing sources engaged more in fact-checking related to the virus [65].

Based on this literature we propose our next hypothesis:

**Hypothesis** **3 (H3).** *Higher conservative media use will be associated with lower willingness to perform recommended COVID-19 related health behavior*.

**Figure 1:**
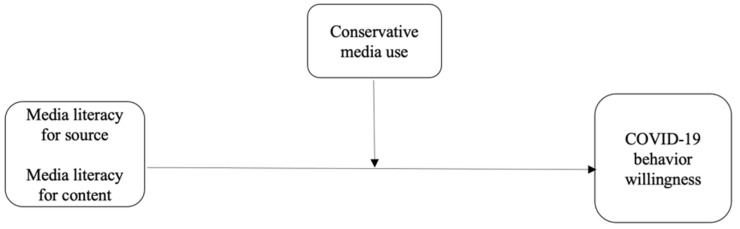
The conceptual model.

Will individuals who are able to think critically about the content and source of information be able to mitigate some of the impact of information from sources such as FOX news that continuously underestimated the pandemic by calling it a hoax (e.g., [19])? To find out, we ask the following research question. Our theoretical model is represented in Figure 1, representing the hypotheses and research question.

RQ1. Will the positive association between conservative media use and willingness to perform recommended COVID-19 related health behavior be moderated by conservative media use?

## 2. Materials and Methods

To test our hypotheses and answer our research questions we conducted a cross-sectional online survey through Qualtrics between 22 June and 18 July 2020. The data was collected after the study was declared exempt by the Institutional Review Board of Washington State University’s (protocol number: 18213). The sample was comprised of 1264 participants total, all of whom were at least 18 years-old. The survey included an over-sample of residents from the state on Washington for a different study (N = 416), as well as demographic and regional quotas. These quotas were based on the 2019 U.S. census.

Quality check measures were used to exclude duplicate respondents, speeders (i.e., respondents randomly answering questions to complete the survey as quickly as possible), and participants who responded in patterns or provided illogical responses (e.g., improper responses to attention check questions, directly contradictory responses to reverse worded questions). Nine respondents were eliminated based on these quality checks. Qualtrics’ quality-control measures excluded poor-quality respondents before providing the data to the researchers. Before analyses were conducted, sample weights were used to make adjustments for the oversample of Washington state residents and to replicate quota samples from the four U.S. regions similar to the 2019 census [66,67].

### 2.1. Measures

#### 2.1.1. COVID-19 Behavior Willingness

Covid behavior willingness was captured with seven items on a 7-point Likert scale (0 = strongly disagree to 6 = strongly agree). Participants were asked to respond to the question “How willing are you to take the following actions? “Regularly and thoroughly clean your hands with an alcohol—based hand rub or wash them with soap and water”, “Maintain at least 6 feet distance between yourself and anyone who is coughing or sneezing”, “Avoid touching eyes, nose and mouth”, “Cover your mouth and nose with your bent elbow or tissue when you cough or sneeze”, “Stay at home if you begin to feel unwell, even with mild symptoms such as headache and slight runny nose, until you recover”, “If you have fever, cough and difficulty breathing, seek medical care promptly”, “Avoid large gatherings”, and “Wear a mask in stores or public gatherings”. The seven items were used to create an index (α = 0.94, M = 4.59, SD = 1.46).

#### 2.1.2. Media Literacy for Source

Similarly, media literacy for source was adapted from prior research [43] and was captured with the help of six items. Using a 5-point Likert scale (0 = strongly disagree to 4 = strongly agree), participants responded to the following items: “I think about how someone creates news that I see”, “I think about who created the news I am seeing”, “I think about what the creator of the news message wants me to think”, “I think about what the creator of the news I am seeing is trying to accomplish”, “I compare news information from different media sources”, and “I check to see if the original source of information I see in the news is clearly stated”. These items were used to create an index (α = 0.90, M = 2.43, SD = 0.97).

#### 2.1.3. Media Literacy for Content

Media Literacy for content was adapted from past research [43] and was measured with the help of five items. Participants responded to a 5-point Likert scale (0 = strongly disagree to 4 = strongly agree) based on the following items: “I compare new information I see in news with other information I have seen before I accept it as believable”, “I look for more information before I believe something I see in news”, “It is important to think twice about what news messages say”, “I often consider whether a message in news is accurate”, “I check on whether information I see in the news is up to date”. These items were used to create an index (α = 0.88, M = 2.69, SD = 0.93).

#### 2.1.4. Conservative Media Use

Conservative media use was measured with three items on a 5-point Likert scale (0 = never to 4 = multiple times a day). The items included “The Fox News cable news channel, website or app from the Fox News cable news organization”, “Conservative Talk Radio website or app (such as The Rush Limbaugh Show)”, “Conservative sources (such as The American Spectator, Breitbart, the Blaze, the Daily Caller, or the Daily Mail)”. These items were used to create an index (α = 0.79, M = 1.23, SD = 1.12).

### 2.2. Controls

We added six variables that are commonly included as controls for these analyses, [68,69,70]. Age (M = 1.55, SD = 1.52) was measured with a single item “Which age group describes you?”(with age groups 18–29 with 27.7%, 30–39/40–49 with 42%, 50–59/60–69 with 23.8% and 70 or over with 6.5%); gender (M = 0.48, SD = 0.50) was measured with the item “How would you describe your gender?”(female with 49.7% and male 48.7%); race (M = 0.51, SD = 0.50) was measured with “How do you describe your ethnicity”; education (M = 3.33, SD = 2.12) was captured with “Please indicate the highest level of education you have completed”; income (M = 3.86, SD = 2.10) was measured with “Which of these describes your household income for 2019?”; and party ID (M = 4.16, SD = 2.17) was measured with an eight-point Likert scale (1 = strong Republican to 8 = Other) “Which of the following best describes your party affiliation”.

## 3. Results

We used process macro (model 1) to conduct the analysis [71]. The first model examined the associations among media literacy for source and conservative media use on COVID-19 behavior. The findings show that the overall model was significant F (9, 1224) = 45.24, *p* < 0.001, R^2^ = 0.25. Among the control variables age, income, race, party ID, and gender were significantly associated with COVID-19 behavior, such that older, higher income, Democrat-identifying female respondents were more willing to follow COVID-19 behavior protocols. The main effect of media literacy for source was significant such that media literacy for source was positively related to COVID-19 behavior (b = 0.47, t (1224) = 10.32, *p* < 0.001; LLCI 0.3811 to ULCI 0.5600), thus providing support for H1. The main effect of conservative media use was also significant such that conservative media use was negatively associated with COVID-19 behavior (b = −0.43, t (1224) = −4.20, *p* < 0.001; LLCI −0.6264 to ULCI −0.2273).

**Figure 2:**
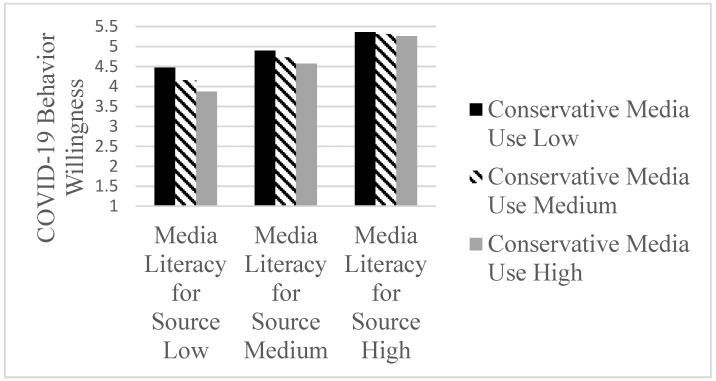
Interaction effects between Conservative Media use and Media Literacy for Source on COVID-19 Behavior Willingness.

The interaction between media literacy for source and conservative media use was shown to be significant (b = 0.11, t (1224) = 3.27, *p* < 0.001; LLCI 0.0438 to ULCI 0.1752). The conditional effects (Table 1) of media literacy for source on COVID-19 behavior are higher for participants with lower conservative media use by one standard deviation below the mean (b = 0.47, t (1224) = 10.32, *p* < 0.001) compared to those at the mean (b = 0.59, t (1224) = 15.40, *p* < 0.001) and above the mean (b = 0.71, t (1224) = 11.86, *p* < 0.001). Specifically, the interaction model revealed that the negative role of conservative media use was highest for the participants with lower media literacy for source (Figure 2).

We conducted a second model to test the relationships among media literacy for content, conservative media use, and COVID-19 behavior. The findings show that the overall model was significant F (9, 1224) = 65.43, *p* < 0.001, R2 = 0.33. Among the control variables age, income, party ID, and gender were significantly associated with COVID-19 behavior, such that older, higher income, Democrat-identifying females were more willing to follow COVID-19 behavior protocols. The main effect of media literacy for content was significant such that media literacy for source was positively related to COVID-19 behavior (b = 0.64, t (1224) = 13.82, *p* < 0.001; LLCI 0.5485 to ULCI 0.7299), in support of H2. The main effect of conservative media use was also significant such that conservative media use was negatively associated with COVID-19 behavior (b = −0.38, t (1224) = −3.55, *p* < 0.001; LLCI −0.5905 to ULCI −0.1704), in support of H3.

**Figure 3:**
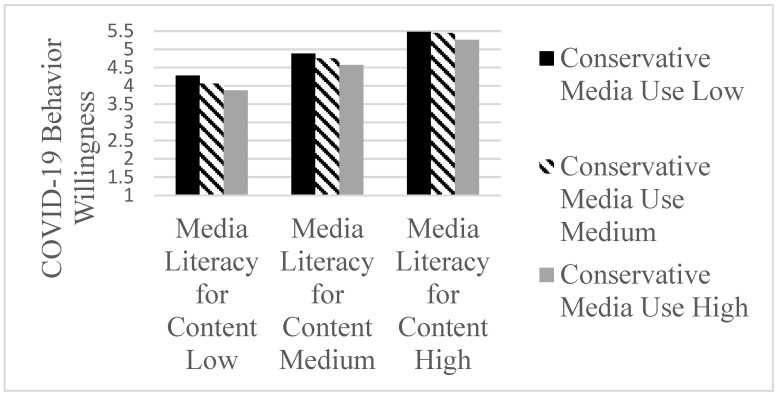
Interaction effects between Conservative Media use and Media Literacy for Content on COVID-19 Behavior Willingness.

The interaction between media literacy for content and conservative media use was shown to be significant (b = 0.10, t (1224) = 2.83, *p* < 0.001; LLCI 0.0293 to ULCI 0.1610). The conditional effects (Table 2) of media literacy for content on COVID-19 behavior are higher for participants with lower conservative media use by one standard deviation below the mean (b = 0.64, t (1224) = 13.82, *p* < 0.001) compared to those at the mean (b = 0.74, t (1224) = 19.96, *p* < 0.001) and above the mean (b = 0.85, t (1224) = 14.58, *p* < 0.001). Specifically, the interaction model revealed that the negative role of conservative media use was highest for the participants with lower media literacy for source (Figure 3).

## 4. Discussion

Using national survey data from the U.S., we examined the relationships between media literacy variables and willingness to perform recommended COVID-19-related health behavior. Media literacy variables have been shown to play a role in health-related attitudes and behaviors [31,32]. Considering the politicization of the pandemic and its politicized coverage on conservative networks (e.g., [19,20]), testing the moderating role of conservative media on the relationship between media literacy and behaviors aimed at mitigating the impact of COVID-19 was critical. Our findings show that conservative media use was negatively associated with these protective behaviors, and that both media literacy variables were positively related with willingness to perform recommended COVID-19 related health behavior.

These findings show that higher conservative media use may have made a difference to how people dealt with the recommended health behaviors for the pandemic. Previous research has shown conditional evidence that, at least among those who already hold rigid political views, consumption of partisan media is associated with polarized attitudes [72]. However, recent studies on the intersection between conservative media use and COVID-19 suggest that the former has been associated with less adherence to stay-at-home orders [56,58], less belief in the severity of being infected by COVID-19 [57], greater misperceptions about the virus [73], and a resistance to mask-wearing [61]. Furthermore, work by [57] finds a similar effect of former President Trump’s public statements downplaying the severity of the virus. As some have noted, there has been considerable overlap between Trump’s public statements and conservative media outlets (namely Fox News) in how the pandemic has been discussed [74], and consumption of this Trump-friendly conservative media has been found to be associated with COVID-19-related misinformation [75]. These studies point to conservative media’s negative associations with evidence-based information about COVID-19 and recommended behaviors for mitigating risk of infection/spread. Specifically, the present study indicates that conservative media use was negatively associated with proactive COVID-19 behavior in both of the models shown here. These results would seem to add further support to prior findings showing that consumption of Fox News programming and other conservative media are associated with less social distancing and less adherence to mask-wearing [56,59].

The role of media literacy is clear in our results. Both media literacy variables demonstrate a positive role in willingness to adopt recommended COVID-19 health behavior. Both media literacy for source and for content had a positive association with behaviors meant to mitigate the effects of COVID-19. These findings echo previous work on self-perceived media literacy [64] and related constructs like media locus of control or need for cognition [41] being associated with fewer misperceptions about COVID-19, some of which include beliefs about the efficacy of the kinds of protective health behaviors examined in the present study. The present study also builds upon previous work on media literacy for source/content and its relation to COVID-19 [40], which found conditional effects for media literacy for content on COVID-19 misperceptions, i.e., liberals higher in media literacy for content were more likely to have reduced misperceptions about COVID-19, unlike conservatives who were higher in these same misperceptions regardless of their media literacy for content levels. In contrast to this previous study which found no direct relationship between media literacy for source/content on COVID-19 misperceptions, the present study found that media literacy for source/content did have a significant main effect on COVID-19 health behavior. Although our findings show a negative association between conservative media use and COVID-19 health behavior willingness, the findings from the interaction effects are critical in a highly politicized pandemic. Even as some respondents consumed a great deal of conservative media, those who could critically think about the source and nature of the information they consumed were still willing to follow the recommended behaviors. Our findings have direct implications for public health. These results not only verify the importance of media literacy in health behavior, which has been shown in past literature [76,77,78], but also demonstrate that media literacy may be able to mitigate the influence of information from sources such as a conservative media, which politicized the pandemic and regularly featured commentators who were skeptical about the potency of the virus [19,20]. We recommend that media literacy should be made compulsory from an early age in schools and colleges, so that individuals are able to critically think about and analyze the information they receive from the media. These skills could not be more important now at a time when the information environment is full of mis- and disinformation, and health information is regularly politicized.

As with all research our study comes with some caveats. Our findings are from a cross sectional survey, so these results cannot make any causal claims. Future research should examine these relationships with panel data or experimental designs. Given the cross-sectional nature of the survey data used here, it is also worth noting that the results presented here could, in part, be a feature of the unique approach to public health pronouncements that the Trump administration took during the time this data was collected. As previously stated, statements about COVID-19 by elected officials and other political elites have been shown to have a significant impact on the attitudes, beliefs, and behaviors of the public [51,53]. Future research should explore the impact of these public pronouncements over time and across different presidential administrations to further understand their role in outcomes related to the pandemic. Furthermore, we only examine two media literacy variables in the present study. Future research should examine other forms of literacy, such as digital information or science literacy. Lastly, our study does not examine actual literacy skills or knowledge structures, which may be important for these associations. Future research should delve deeper into these relationships.

## 5. Conclusions

Despite some of these limitations, our study examines a critical relationship during a deeply politized pandemic. At present, even as the impact of the Omicron variant of COVID-19 starts to show signs of decline, the most recent wave of the pandemic has now resulted in a greater loss of life in the U.S. than the Delta wave [79]. These recent figures alone speak to the importance of credible, actionable information about the pandemic and behavioral guidelines from public health institutions. The present study further contributes to trends in the existing literature that signal a link between consumption of conservative media and less willingness to adopt best practices for mitigating the risks of COVID-19. However, critical thinking about the source and content of media messages are shown here to be positively associated with recommended behavioral health practices for COVID-19, and furthermore show signs of blunting conservative media’s association with less frequent adoption of these same practices. As such, the importance of media literacy’s role in the pandemic should be recognized by scholars and public health professionals alike—not only for its association with adoption of recommended behavioral health practices, but for its potential role in depoliticizing what has become another deeply divisive issue among many citizens.

## Figures and Tables

**Table 1:** Conditional Effects of Conservative Media Use and Media Literacy for Source on COVID-19 Behavioral Willingness.

	Conservative Media Use	*t*	LLCI	ULCI
COVID-19 Behavior Willingness	−1 SD	10.3180 ***	0.3811	0.5600
	M	15.4003 ***	0.5140	0.6641
	+1 SD	11.8592 ***	0.5943	0.8299

*** *p* < 0.001.

**Table 2:** Conditional Effects of Conservative Media Use and Media Literacy for Content on COVID-19 Behavioral Willingness.

	Conservative Media Use	*t*	LLCI	ULCI
COVID-19 Behavior Willingness	−1 SD	13.8233 ***	0.5485	0.7299
	M	19.9609 ***	0.6693	0.8152
	+1 SD	14.5798 ***	0.7349	0.9634

*** *p* < 0.001.

## Data Availability

Data can be available upon request.
